# Why do habitual violent video game players believe in the cathartic effects of violent video games? A misinterpretation of mood improvement as a reduction in aggressive feelings

**DOI:** 10.1002/ab.22005

**Published:** 2021-11-06

**Authors:** Riccarda Kersten, Tobias Greitemeyer

**Affiliations:** ^1^ Social Psychology University of Innsbruck Innrain 2 Innsbruck Austria

**Keywords:** aggressive feelings, catharsis, experience sampling method, mood, violent video games

## Abstract

Previous research found that violent video game play leads to increased aggression, but many people (mainly habitual violent video game players) still believe that playing violent games releases aggressive feelings and in turn reduces aggressive behavior. Other research has shown that video game play can have a positive impact on the player's mood. Based on the General Aggression Model and mood management theory, we thus hypothesized that habitual violent video game players misinterpret their better mood after game play as a reduction of aggressive feelings and hence believe in the cathartic effects of violent video games. Two studies examined this reasoning in the player's natural habitat. Habitual video game players were surveyed multiple times for a period of 2 weeks before and after each gaming session. Results showed that playing video games improved the participant's mood, which in turn was positively associated with the belief in the cathartic effect of violent video game play. Importantly, this relation held when controlling for the player's actual level of aggressive feelings. Study 1 further showed that playing a violent game tended to lead to a higher level of reported aggressive feelings after playing. In contrast, in Study 2, level of reported aggressive feelings was not related to the violence of the game. Taken together, habitual violent video game players (erroneously) believe in the cathartic effects of violent video games, because they are in a better mood after playing.

## 1 INTRODUCTION

Since the first video games emerged in the 1970s, the popularity of virtual games has continuously risen (Gough, 2019). For many years, researchers have been examining the impact of video game play on behavior, affect, and cognition. While some studies have highlighted possible benefits of video games (for reviews, Granic et al., 2014; Greitemeyer, 2022), playing video games (in particular, violent video games) also has negative effects, such as a reduction in cognitive control (Bailey et al., 2010; Hummer et al., 2019; West et al., 2020) and an increase in aggression. In fact, a vast number of studies showed that playing violent video games increases aggressive behavior and related cognition and affect (for meta‐analyses, Anderson et al., 2010; Greitemeyer & Mügge, 2014; Prescott et al., 2018). Nevertheless, drawing upon the concept of catharsis, many video game players still firmly believe that playing violent games cleans from aggressive feelings. The general cathartic hypothesis was rejected decades ago (Geen & Quanty, 1977) and contemporary research has reached the same conclusion. Nonetheless, the idea that venting aggression in video games reduces aggressive feelings and behaviors continues to enjoy widespread public support (Gentile, 2013; Greitemeyer, 2014).

While there is no empirical support for the cathartic effects of violent video game play, video games may have beneficial impacts on the player's affective states. Indeed, studies have shown that playing video games has a positive impact on the player's mood (e.g., Bowman & Tamborini, 2015; Fleming & Rickwood, 2001; Russoniello et al., 2009). Importantly, however, the idea of catharsis is about a decrease in aggressive feelings and in turn aggressive behavior; catharsis does not imply being in a positive mood (Gentile, 2013). In the present research, we test the idea that because playing violent video games improves the player's mood, players (erroneously) believe that violent video games lead to a reduced level of aggressive feelings.

We further examined whether the impact of an improved mood after playing a violent video game on the belief that violent video game play reduces aggressive feelings holds when controlling for the player's actual level of reported aggressive feelings. Previous research has shown that aggression more generally (not only media violence) is often a pleasant experience and that the perception of improved affective states after behaving aggressively fuels further aggression (Chester, 2017). For example, Bushman et al. (1999) showed that individuals who were inclined to enjoy acting out their aggression were the most aggressive in a subsequent social encounter. Hence, violent video game players who particularly enjoy performing virtual violence should also exhibit the most aggressive feelings after they had played a violent video game. Therefore, we predicted that the impact of the player's mood change on the belief in the cathartic effect would hold when controlling for the player's actual level of aggressive feelings. To examine these predictions, we surveyed habitual video game players in their own natural habitat multiple times before and after playing video games.

### 1.1 Theoretical perspectives

#### 1.1.1 Mood management theory

According to mood management theory (Zillmann, 1988), the consumption of entertaining messages often serves the regulation of mood states. The theory's basic assumptions are that individuals strive to get rid of negative moods and maintain and intensify good moods. Mood management theory further assumes that the experience that a random arrangement of environmental stimuli leads to mood enhancement leaves a memory trace that increases the likelihood for making similar arrangements under similar conditions in the future (Zillmann, 2000). Zillmann (1988) argues that, whereas formerly it was necessary to go fishing, climbing, or playing tennis to raise one's mood, in modern times individuals do no longer have to move themselves to desired environments, but they move the environments instead. For example, individuals learn by operant conditioning which video games improve their mood and are likely to select these again in the future. Based on these theoretical assumptions, we reasoned that avid violent video game players choose those games that actually improve their moods.

#### 1.1.2 Catharsis hypothesis

The concept of catharsis traces back to the ancient Greek idea and was later suggested by Sigmund Freud and Josef Breuer as a treatment for hysteria. By experiencing and expressing repressed emotions, symptoms of psychological diseases were believed to be alleviated. A more recent definition of catharsis has been given by Geen and Quanty (1977) who define aggression catharsis as a hypothesized process which follows aggression and that is postulated to lead to a reduction in aggressiveness. They argue that the reduction of aggressiveness is not necessarily a product of catharsis as it is intended, but could also be a byproduct of other processes. The simple passage of time, for example, often accounts for a decrease in aggression. The aggression catharsis hypothesis in relation to media effects suggests that virtual violent content is watched and thereby reduces the viewer's aggressive feelings. Gentile (2013) defines the aggression catharsis hypothesis as: “a common belief that playing violent video games or watching violent TV/movies allows people to ‘vent' their aggressive inclinations and therefore behave less aggressively after playing/watching” (p. 492). Advocates of the aggression catharsis hypothesis argue that while playing violent video games, aggressive feelings can be expressed and are, therefore, reduced afterward.

#### 1.1.3 The General Aggression Model

With the General Aggression Model (GAM), Anderson and Bushman (2002, 2018) postulated a theoretical framework that explains why exposure to media violence leads to more aggression instead of less aggression as postulated by catharsis hypothesis. The authors describe aggression as a behavior that is directed toward other individuals and intends to cause harm, to injure, or to irritate another person (cf. Huesmann, 1998). The GAM assumes that knowledge structures, which develop based on experiences, have an impact on a person's reactions and can become automatized with use. Furthermore, Anderson and Bushman (2002) argue that these knowledge structures can be linked to affective states, behavioral programs, or beliefs and are in turn used to guide people's interpretations and behavioral responses to environmental events. When a knowledge structure that contains anger is activated, anger will be experienced and in some circumstances actually expressed. Based on these assumptions, the GAM sets out three factors relevant for the occurrence and expression of anger: (1) person and situation inputs, (2) cognitive, affective, and arousal routes, which define the processing and the impacts of the input variables, and (3) outcomes of these processes.

Situational factors include variables such as aggressive cues, provocation, or frustration. The present research examines the impacts of aggressive cues in the form of violent video games on aggressive feelings. As personal factors, Anderson and Bushman (2002) name traits, sex, beliefs, attitudes, values, long‐term goals, and scripts. They argue that certain traits make individuals more likely to exhibit higher levels of aggression. To account for personal differences in the present research, participant sex was considered as a control variable. These input variables can lead to outcome changes by taking cognitive or affective routes or by causing changes in arousal levels. Changes of internal states affect appraisal and decision processes, which in turn may lead to either thoughtful or impulsive actions.

In line with the GAM, we assumed violent video game play to lead to higher aggressive feelings after playing. However, it may also be that when experiencing aggressive impulses, players are particularly likely to choose a violent game. That is, we hypothesize a positive relationship between the game's violent content and the player's level of aggressive feelings before and/or after playing. A positive relationship before playing would be in line with the selection hypothesis that highly aggressive individuals are particularly likely to seek out violent media contents (Huesmann et al., 2003). It would be also in line with the finding that video game players use games to regulate their emotions (for reviews, Hemenover & Bowman, 2018; Villani et al., 2018). A positive relationship after playing would be in line with the socialization hypothesis that violent video game exposure causes the user to experience more aggressive feelings (Anderson et al., 2007). Notably, the selection and socialization hypotheses are by no means mutually exclusive. However, if a person chooses to play a violent video game when experiencing highly aggressive impulses, there is little room for this person to feel even more aggressive (due to a ceiling effect). Hence, we anticipated an overall positive relationship between violent video game play and the player's level of aggressive feelings, but we were unsure about whether this relationship would be reliable for both, before and after playing.

### 1.2 Previous empirical studies on violent video games, aggression, and the player's mood

#### 1.2.1 The impact of violent video games on aggression

Dozens of studies and several meta‐analyses investigated the relationship between violent video game play and aggression. A recent meta‐analysis of longitudinal studies published by Prescott et al. (2018, 2018) found that overall video game violence was positively related to aggressive behavior. Greitemeyer and Mügge (2014) conducted a meta‐analysis of experimental, correlational, and longitudinal studies and showed that exposure to violent video games increases aggression and decreases prosocial outcomes. Anderson et al. (2010) concluded, based on the most comprehensive meta‐analysis of experimental, cross‐sectional, and longitudinal studies so far, that violent video game play represents a causal risk factor for aggression occurring in the form of aggressive behavior, aggressive cognition, and aggressive affect, as well as decreased empathy and lowered prosocial behavior. On the other hand, some studies did not find significant effects (e.g., Kühn et al., 2019; McCarthy et al., 2016). However, given that the typical effect of violent video games on aggression is not large, it is to be expected that not all studies reveal significant effects.

#### 1.2.2 The impact of violent video games on the player's mood

Various studies have shown that video games of different genres can improve the players' mood (Bowman & Tamborini, 2015; Fleming & Rickwood, 2001; Russoniello et al., 2009). For example, Fleming and Rickwood (2001) found in an experimental setting that positive mood increased significantly among children after playing a violent video game. A mood‐enhancing effect of playing a violent video game was also evident in a study by Greitemeyer et al. (2019) that revealed a significant correlation between everyday sadism and the mood‐enhancing effect of playing a violent video game. A recent meta‐analysis of qualitative, cross‐sectional, and experimental studies summarized the impact of video game play on mood and emotion regulation (Villani et al., 2018). Overall, results were in line with mood management theory (Zillmann, 1988), in that, when the selection of videogames was coherent with users' needs and satisfied the needs for competence and autonomy, playing led to enjoyment and mood repair, which, in turn, led to a reduction of negative feelings.

#### 1.2.3 Aggression catharsis

Additionally to the many studies that found aggression‐increasing effects of violent video games (see above), several meta‐analyses revealed effects strongly contradicting the cathartic effects of media violence in general. Allen et al. (1995) found that media depiction of violent sexual activity generates more aggression with a small to medium effect size. According to Gentile (2013), almost all previous meta‐analyses found that media violence heightens aggressive thoughts, feelings, and behavior with a small to moderate effect size and he argues: “Taken together, it appears that there is no possible way that the aggression catharsis hypothesis can be accurate. It nevertheless continues to “feel correct at a phenomenological level” (p. 491). Although since the 19th century a lot of empirical evidence contradicting the cathartic effects of media violence arose, it still is a common belief that expressing and deflating anger results in a less angry state. For example, a study by Greitemeyer (2014) showed that habitual violent video game players were more likely to believe that playing violent games decreases the player's aggression than were individuals who do not play violent video games. A possible explanation for this could be that people who like to watch media violence reduce cognitive dissonance by believing in the cathartic effects of violent media (Gentile, 2013). Furthermore, Bushman et al. (2001) showed that people engage in aggressive behavior because they believe that it will change their bad mood. Indeed, as many studies showed (see above), playing a self‐chosen (violent) video game improves the player's mood. This mood improvement may lead people to believe that they would behave less aggressively when in fact aggressive behavior is even more likely to appear (Gentile, 2013). Therefore, it will be examined whether a mood improvement after playing accounts for the belief in the cathartic effect of violent video games. Addressing this hypothesis has important theoretical implications for the validity of the GAM (and related theories) on the one hand, and aggression catharsis on the other.

### 1.3 The present research

#### 1.3.1 Methodological approach

To test our hypotheses, we ran two experience sampling studies. Experience sampling methods (ESMs) go along with multiple benefits: they allow the examination of reported events and impacts of these events in natural and spontaneous contexts. Data collection can take place within the participant's own habitat and thus enhances the external validity of results (Moreno et al., 2012). Moreover, experience sampling reduces the likelihood of mistakes due to retrospection, as the amount of time between an experience and the assessment of the experience is minimized (Bolger et al., 2003). Diary methods, Bolger et al. (2003) argue, offer the possibility to examine antecedents, correlates, and consequences of daily experiences or events. Furthermore, they can be used to investigate individual differences in these processes. Thus, diary methods respectively ESMs represent a sensible extension to experimental studies examining the causal impact of video game play under laboratory conditions.

#### 1.3.2 Hypotheses

As outlined above, we assumed that because playing a violent video game improves the player's mood, they erroneously believe that violent video game play decreases aggression. That is, we hypothesized that the improved mood, which the habitual video game player experiences after playing a violent game, is positively associated with the belief in the cathartic effect of violent video game play (H1). We thus postulate a two‐way interaction between a better mood after playing and game violence on the belief in cathartic effects.

We further assumed that participants that play violent video games would report higher levels of aggressive feelings before and/or after playing compared to participants that played video games with little or no violent content (H2). Please note that we focused on the player's level of aggressive feelings because catharsis hypothesis postulates that venting aggression in violent video game play cleans from aggressive feelings, which in turn leads to reduced aggressive behavior. We will return to the issue of how we measured aggressive feelings in the General Discussion. Please also note that we relied on habitual video game players as participants. That is, individuals who do not play video games could not participate in our studies. This is important insofar as the range of the video game violence exposure scores is decreased, which in turn is likely to reduce the relationship between video game violence and aggressive feelings. In sum, our set of studies provides a conservative test of the hypothesis that playing violent video games is associated with increased levels of aggressive feelings.

In Study 2, we additionally asked participants to indicate on a dichotomous scale whether the game they played was violent or not and examined whether the impact of the participant's improved mood on the belief in the cathartic effect would hold when controlling for the participant's actual level of aggressive feelings. As experienced positive affect after behaving aggressively is an important cause of future aggression (Bushman et al., 1999; Chester, 2017), we hypothesized that the impact of the participant's better mood on the belief in the cathartic effect of violent video game play would remain significant when controlling for the participant's level of aggressive feelings (H3). Ethical approval was given by the university where the studies were carried out. The questionnaires and data for both studies are openly available in the Open Science Framework: DOI 10.17605/OSF.IO/6CRX4.

## 2 STUDY 1

### 2.1 Method

Previous results suggest that people who play violent video games tend to deny the aggression increasing effects of violent video games (Gentile, 2013; Greitemeyer, 2014). To avoid distortion, participants were told that the study was about the effects of video games on mood, whereas we did not refer to any aggression‐related outcome. Among all participants, five 100€ coupons were raffled. The same applies to Study 2. Psychology students additionally received course credit for participating.

#### 2.1.1 Participants

Participants were recruited in university classes, via a university mailing list, and via social media. Requirements were that participants were full‐aged and that they habitually played video games. Gamers of all genres were invited to participate, and games could be played on various devices. Due to the ESM design, no power analyses were conducted to estimate the required sample size. Instead, a period of 3 months was determined for recruitment with the aim of running as many participants as possible. Two‐hundred and fifty‐eight individuals completed the initial survey. Of these, 73 were female, 183 male, and 2 diverse. Mean age was 23.6 years (*SD* = 4.4). The majority of the sample was represented by students, 194, 75.2%, compared with nonstudents, 64, 24.8%. Among the 258 people who participated in the initial survey, 181 participants filled out at least two questionnaires in the playing phase and of these, 156 completed the final survey. Overall, 1657 “before”‐questionnaires and 1790 “after”‐questionnaires were filled out during the 2‐week playing phase. Questionnaires after gaming sessions were excluded when participants indicated that a time span of more than 5 min had passed since the last gaming session. The remaining questionnaires were matched, that is, each “before”‐questionnaire was only included when a congruent “after”‐questionnaire was filled out and vice versa. All other questionnaires were excluded from the analyses. Additionally, we ensured that the timestamp of the before and after questionnaires matched with the participants' information on how long they played. The maximum number of questionnaires filled out in the playing phase was 54 questionnaires, which is equivalent to 27 sessions. On average, participants completed a mean of *M* = 12.31 questionnaires during the playing phase.

#### 2.1.2 Measures

The data assessment included three phases. Initially, participants provided sociodemographic data such as sex, age, country of origin, education level, and current occupation in a one‐time assessment. Afterward, habitual video game play was assessed. To this end, participants indicated their three favorite video games and rated each of them regarding difficulty, pleasure, fun, and level of violence, as well as level of competition on a scale from 1 (*very low*) to 9 (*very high*). Participants also indicated how often they play each game ranging from 1 (*sometimes*) to 7 (*very often*). As in previous research (e.g., Anderson et al., 2007), to assess habitual violent video game play, we multiplied each game's level of violence by the playing frequency and summed these values over the three games. Furthermore, trait aggression was assessed. To this end, the short trait aggression scale by Bryant and Smith (2001), which consists of 12 items (*α* = 0.80; sample item: “Sometimes I fly off the handle for no good reason”) was employed. At the end of the initial survey, participants were asked for their email addresses so that further questionnaires as well as reminder emails could be sent. To preserve their anonymity, a double opt‐in procedure was implemented.

The second phase of the study started directly after the initial questionnaire was filled out. Participants were asked to complete a questionnaire before and after every gaming session for a period of 2 weeks. No minimum length or frequency of gaming sessions was set and various electronic devices could be used for playing. Immediately after filling out the initial survey, participants received an email with two links to questionnaires, as well as information about the further procedure. One link led to the questionnaire that participants were asked to fill out before every video game session in the following 2 weeks. The other link provided the questionnaire that participants were requested to fill out after each gaming session. Participants received a detailed description about how to save the links and were instructed that it is important to complete the questionnaires immediately before and after every gaming session with a maximum delay of 5 min. Additionally, participants obtained a reminder email every day. Both questionnaires (before and after playing) included the same measurements of current mood and aggressive feelings. To assess the participant's improved mood, participants indicated how they feel at the present moment on a scale from 1 (*very bad mood*) to 9 (*very good mood*) and how satisfied they are, ranging from 1 (*very unsatisfied*) to 9 (*very satisfied*). Items were summarized–one for mood before (*α* = 0.92) and one for mood after playing (*α* = 0.94). With regard to measuring participant's aggressive feelings, we felt it important that the items would not overlap with the items which we used to measure bad mood. Therefore, items were chosen that differed from those assessed in the Positive and Negative Affect Scale (PANAS, Watson et al., 1988; e.g., upset, alert, distressed). On the other hand, the items should not be too transparent measures of aggressive feelings. Therefore, items such as “aggressive” or “combative” were avoided. In the end, the items irritated, troubled, heated, offended, and upset, as well as indulgent and peaceful as reversed items were chosen from the Hostile Affect Scale by Anderson et al. (1995). Participants were asked to indicate how well these items describe how they currently feel on a scale from 1 (*strongly disagree*) to 5 (*strongly agree*). Items were summarized to an aggressive feelings factor before (*α* = 0.87) and after playing (*α* = 0.87). Participants were also asked how many minutes passed since they ended the last video game session and the name of the lastly played video game and the duration of play were assessed. Furthermore, after playing, participants rated the game regarding difficulty, pleasure, fun, and level of violence, as well as level of competition and own achievement on a scale from 1 (*very low*) to 9 (*very high*).

Fifteen days after filling out the initial survey, participants received the link to the final questionnaire via email, which assessed the belief in the cathartic effect. To this end, participants indicated to what extent they believe that playing a violent video game reduces aggression and whether they think that playing a violent video game leads to a purification of aggressive impulses. Both items were assessed on a scale from 1 (*I don't believe at all*) to 9 (*I strongly believe*). Furthermore, participants answered to what extent they think that playing a violent video game decreases or increases aggression on a scale from 1 (*strongly decreases aggression*) to 9 (*strongly increases aggression*). Item 3 was reversed and an overall index was created (*α* = 0.86). These items were successfully employed in previous research (Greitemeyer, 2014). Finally, participants indicated what they thought this study's purpose was. No participant was excluded due to guessing the aim of the present study correctly. The same applies to Study 2.

Because the study was part of a student Master's project, additional measures were employed. For example, we examined whether participants that have high scores on neuroticism, low scores on conscientiousness, and low scores on agreeableness would be particularly likely to play violent video games (they were not). Because these measures are not relevant for the present purposes, we do not report them here.

### 2.2 Results

#### 2.2.1 Attrition analyses

Attrition analyses were performed to examine whether the three samples of individuals who completed only the initial survey, of those who additionally participated in the playing phase, and of individuals who also filled out the final survey significantly differed from each other. One‐way between‐subjects analysis of variances showed marginally significant differences in terms of age, *F*(2, 253) = 3.01, *p* = .051, and habitual violent video game play, *F*(2, 253) = 2.99, *p* = .052. Participants who completed the first survey and took part in the playing phase were older and reported a higher amount of violent video game play, mean age = 25.32, mean *VVG* = 85.45, compared with those who only filled out the initial survey, mean age = 24.09, mean *VVG* = 66.59, and those who participated in all three survey phases, mean age = 23.14, mean *VVG* = 66.96. No significant differences were observed for trait aggression, *F*(2, 253) = 0.14, *p* = .868. Furthermore, a Kruskal–Wallis test showed no significant differences for participant sex, *χ*² (2, 253) = 2.27, *p* = .321.

#### 2.2.2 Preliminary analyses

Previous research has shown that playing preferred video games improves the player's mood (Villani et al., 2018). We were able to replicate these findings. The participant's experienced mood after playing a self‐chosen game was significantly better than before, *t*(142) = 3.15, *β* = 0.16, *p* = .004. Further analyses showed that neither the additive effect of violent content, *t*(608) = −0.73, *β* = −0.02, *p* = .466, nor the interaction effect of level of violence and time, *t*(467) = 1.35, *β* = .02, *p* = .179, were significant, indicating that violent video games led to similar mood improvements as nonviolent video games did.

Descriptive statistics and intercorrelations for the full sample for the variables sex, age, trait aggression, and habitual violent video game play are shown in Tables 1 and 2. No significant correlations among sex, age, violent video game play, and trait aggression were found.

**Table 1:** Means, standard deviations (*SD*), and bivariate correlations (Study 1, initial survey, *N* = 258)

	*M*	*SD*	1	2	3
1 Sex	–	–			
2 Age	23.61	4.42	0.04		
3 Trait aggression	1.87	0.59	0.01	0.08	
4 VVG	68.44	34.42	0.10	−0.10	0.05

*Note*: Participant sex was coded 1 = female, 2 = male.

Abbreviation: VVG, habitual violent video game play.

**Table 2:** Means and standard deviations (Study 1, ESM surveys, *n* = 181; Sessions = 1493)

	*M*	*SD*	Minimum	Maximum
Aggressive feelings				
Before playing	1.84	0.70	1.00	5.00
After playing	1.93	0.75	1.00	5.00
Mood				
Before playing	6.42	1.63	1.00	9.00
After playing	6.58	1.65	1.00	9.00

Abbreviation: ESM, experience sampling method.

#### 2.2.3 Main results

Linear mixed models were calculated and p‐values were derived by Satterthwaite's method. Luke (2017) argues that Satterthwaite approximations are preferable for avoiding Type 1 errors. They were found to produce the most consistent Type 1 error rates and were not overly sensitive to sample size.

Calculations for H1 were conducted on the sample of 156 individuals who participated in all three survey phases. A model with fixed effects for violent content, sex, the interaction of mood before playing and violent content, the interaction of mood after playing and violent content, as well as random intercepts for a session, was created. The outcome variable was the score of believing in the cathartic effect. The predictors violent content, *t*(1440) = 4.12, *β* = 0.18, *p* < .001, sex, *t*(1440) = 7.91, *β* = 0.82, *p* < .001, and the interaction of mood before playing and level of violence, *t*(1440) = −5.36, *β* = −0.03, *p* < .001, were significant. These results indicate that the higher the violent content of the game, the more people believe in the cathartic effects of violent video games. That is, as, in previous research (Greitemeyer, 2014), habitual violent video game players are more inclined to believe in the cathartic effect of violent video game play than are individuals who play video games with little or no violent content. Moreover, sex had a strong impact on believing in catharsis, indicating that males were more likely to believe in the cathartic effect of violent video games.

Most importantly and in line with Hypothesis 1, the interaction of violent content and mood after playing was significant, *t*(1440) = 2.86, *β* = 0.02, *p* = .004.^1^ All parameter estimates are reported in Online Supporting Information and the pattern for the interaction is shown in Table 3. Participants that played a violent video game and were in a better mood after playing were more likely to believe in the cathartic effect of violent video games than those who did not play a violent video game and/or those who were not in a better mood. Overall, H1 received support from the data in that improved mood after violent video game play was positively associated with the belief in the cathartic effect of violent video game play.

**Table 3:** Means and standard deviations (*SD*) (in parentheses) of the belief in catharsis as a function of violent content and the player's level of mood change

	Violent content	
Player's level of mood change	Low	High
Study 1		
Low level	4.10 (1.81)	4.79 (1.89)
High level	4.72 (1.89)	5.65 (1.86)
Study 2		
Low level	4.26 (1.75)	4.98 (1.50)
High level	4.49 (1.62)	5.42 (1.56)

*Note*: Low level of mood change = −1 *SD*, high level of mood change = +1 *SD*.

Study 1: Low violent content = −1 *SD*, High violent content = + 1 *SD*.

To analyze H2, a model was created that included aggressive feelings before and after playing as an outcome variable to analyze whether the predictor variables would have an impact on aggressive feelings on either of both time points. As predictor variables, we included violent content of the game and sex as fixed effects, as well as session as a random effect. The impact of sex, *t*(297) = −0.21, *β* = −0.07, *p* = .037, was significant, indicating that females were more aggressive before and after playing than males. Furthermore, the impact of violent content, *t*(2980) = 2.06, *β* = 0.01, *p* = .040, was significant. As hypothesized, participants that played a violent game reported higher levels of aggressive feelings when both time points were considered simultaneously.

**Figure 1:**
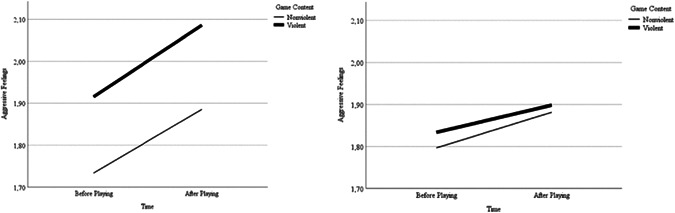
Means of aggressive feelings before and after playing as a function of violent content (left: Study 1, Sessions = 921; *Note*: Nonviolent Game = −1 *SD*, Violent Game: +1 *SD*; right: Study 2, Sessions = 1500). *SD*, standard deviation

Because the additive effect of violent content on aggressive feelings before and after playing was significant, two more models were calculated to examine the impact of violent content on aggressive feelings before and after playing separately. In both analyses, we controlled for the participant's sex and we included random effects for session. Before playing, the effect of level of violence on aggressive feelings was not significant, *t*(1401) = 1.09, *β* = 0.00, *p* = .275. After playing, the effect of level of violence was marginally significant, *t*(1375) = 1.85, *β* = 0.01, *p* = .065. The relation between aggressive feelings before and after playing and the game's violence is visualized in Figure 1.

Furthermore, we examined the change in aggressive feelings between before and after playing a violent game. A baseline model with fixed effects for time and sex, as well as random intercepts for session with random slopes for time was created. The model showed a significant effect of time, *t*(168) = 3.15, *β* = 0.10, *p* = .002, reflecting that participants reported to feel more aggressive after compared to before playing. The effect of sex was marginally significant, *t*(2976) = −1.89, *β* = −0.06, *p* = .059. In contrast, the interaction of time and violent content was not significant, *t*(841) = 0.16, *β* = 0.00, *p* = .873. Further analyses showed that the aggressive feelings increasing effect of playing (any) video game was related to the game's competitive content, *t*(700) = 3.73, *β* = 0.03, *p* < .001, and game achievement, *t*(2209) = −9.97, *β* = −0.10, *p* < .001.

### 2.3 Discussion

As we hypothesized, participants that were in a better mood after playing a violent game were the ones who believe most strongly in the cathartic effects of violent video games. This effect was found independent of sex, a number of gaming sessions, and the player's mood before playing. Although no causal conclusions can be drawn, our results provide first empirical evidence that the habitual violent video game player's improved mood after playing heightens the (erroneous) belief that violent video game play decreases aggression.

Based on previous meta‐analyses (Anderson et al., 2010; Greitemeyer & Mügge, 2014; Prescott et al., 2018), we hypothesized that participants would exhibit more aggressive feelings before and/or after playing if they played a violent video game compared to less violent games. We did find a positive relation between the game's violence and the player's level of aggressive feelings when considering both time points (before and after playing) together. Moreover, the relationship between the game's violence and the player's reported aggressive feelings after playing was positive and marginally significant. These results indicate that playing a violent game tended to lead to a higher level of aggressive feelings after playing. In contrast, the violence of the game was not significantly related to the level of aggressive feelings before playing. Overall, the results were more supportive for the socialization than for the selection hypothesis, in that playing violent video games tended to increase aggressive feelings, whereas aggressive people were not particularly likely to choose a violent game for playing.

## 3 STUDY 2

### 3.1 Method

The procedure and the materials were very similar to Study 1. A few modifications were made, which are described in the following sections. Most importantly, participants indicated on a dichotomous scale whether the game they played was violent or not, which enabled us to test H3 that the impact of the player's mood on the belief in the cathartic effect of violent video game play holds when controlling for the player's level of aggressive feelings.

#### 3.1.1 Participants

We recruited participants in university classes, via a university mailing list, and via social media. Again, a period of 3 months was determined for recruitment with the aim of running at least as many participants as in Study 1. Three‐hundred and forty‐six individuals completed the initial survey (86 female, 258 male, 2 diverse; mean age: 23.0 years, *SD* = 6.0). The majority of participants were students, 299, 86.4%, compared to nonstudents, 47, 13.6%. Among the 346 participants who took part in the initial survey, 222 participants completed at least two questionnaires in the playing phase, and of these, 169 filled out the final survey. Overall, 1962 “before”‐questionnaires and 1749 “after”‐questionnaires were completed during the two‐week playing phase. The maximum number of questionnaires filled out in the playing phase was 54 questionnaires, which equals 27 sessions. On average, participants completed a mean of *M* = 13.54 questionnaires during the playing phase.

#### 3.1.2 Measures

The short trait aggression scale (Bryant & Smith, 2001) showed an internal consistency of *α* = 0.76. Mood before playing (*α* = 0.91) and after playing (*α* = 0.98) showed a similar internal consistency as in Study 1. For the aggressive feelings scales before and after playing, internal consistencies were: *α* = 0.88 and *α* = 0.87. For the belief in catharsis, internal consistency was: *α* = 0.75.

In Study 2, we changed the order of the mood and aggressive feelings items in the before‐ and after‐questionnaires. Most importantly, the second modification was that we asked participants on a dichotomous scale additionally to the continuous scale whether the game they lastly played was violent or not. In the following analyses, if not otherwise specified, we employed the variable violent content on a dichotomous scale (coded: 1 = nonviolent game, 2 = violent game).

### 3.2 Results

#### 3.2.1 Attrition analyses

No significant differences between drop‐out groups were found. The full analyses can be obtained from the first author upon request.

#### 3.2.2 Preliminary analyses

As in Study 1, the player's mood was improved after playing, *t*(154) = 5.47, *β* = 0.28, *p* < 0.001. Again, neither the additive effect of violent content, *t*(825) = −0.91, *β* = −0.01, *p* = .364, nor the interaction with time, *t*(695) = 1.35, *β* = 0.01, *p* = .179, had a significant impact on mood, indicating that nonviolent games led to a similar mood improvement as did violent games.

Descriptive statistics and intercorrelations for the full samples are shown in Tables 4 and 5. In contrast to Study 1 where no significant correlation between violent video game play and trait aggression was found, Study 2 revealed a significant positive relation between habitual violent video game play and trait aggression.

**Table 4:** Means, standard deviations (*SD*), and bivariate correlations (Study 2, initial survey, *N* = 346)

Variable	*M*	*SD*	1	2	3
1 Sex	–	–			
2 Age	23.00	6.01	0.08		
3 Trait aggression	1.80	0.51	−0.09	0.00	
4 VVG	69.60	32.77	0.10	−0.02	0.13*

*Note*: Participant sex was coded 1 = female, 2 = male.

Abbreviation: VVG, habitual violent video game play.

* *p* < .05.

**Table 5:** Means and standard deviations (Study 2, ESM surveys, *n* = 222; Sessions = 1500)

Variable	*M*	*SD*	Minimum	Maximum
Aggressive feelings				
Before playing	1.81	0.70	1.00	4.57
After playing	1.89	0.72	1.00	5.00
Mood				
Before playing	6.30	1.65	1.00	9.00
After playing	6.58	1.56	1.00	9.00

Abbreviation: ESM, experience sampling method.

#### 3.2.3 Main results

Examining H1, we conducted our analysis on the sample of the 169 participants that completed all three survey phases. As is Study 1, we created a model that included the belief in catharsis as an outcome variable, as well as violent content and sex as fixed predictors and session as a random predictor. Furthermore, the interaction of mood before playing and violent content as well as the interaction of mood after playing and violent content were included in the model. Unlike in Study 1, the predictors violent content, *t*(1360) = 0.19, *β* = 0.01, *p* = .848, the interaction of mood before playing and level of violence, *t*(1353) = −0.32, *β* = 0.00, *p* = .750, as well as sex, *t*(1361) = −0.92, *β* = −0.09, *p* = .356, did not have an impact on believing in catharsis. However, and most importantly, the interaction of violent content and mood after playing had a significant impact on the belief in the cathartic effect, *t*(1350) = 3.89, *β* = 0.02, *p* < .001. All parameter estimates are reported in the supplementary material and the pattern for the interaction is shown in Table 3. In line with H1, participants that played a violent game and were in a better mood were the ones who believe most strongly in the cathartic effect of violent video game play.^2^

Examining H2, we analyzed all participants that completed at least two questionnaires during the playing phase. Similar to Study 1, we firstly examined the effect of violent content on aggressive feelings before and after playing under consideration of sex and random intercepts for session. Neither the impact of sex, *t*(2995) = 1.45, *β* = 0.04, *p* = .149, nor of violent content, *t*(2995) = −0.51, *β* = −0.01, *p* = .611, were significant. Furthermore, we created two models analyzing the impact of violent content on aggressive feelings before and after playing separately. When including sex as a fixed and session as a random predictor, neither violent content, *t*(1496) = 0.318, *β* = 0.01, *p* = .750, nor sex, *t*(1447) = −0.79, *β* = −0.33, *p* = .430, had a significant influence on aggressive feelings before playing. However, sex, *t*(1480) = 2.80, *β* = 0.12, *p* = .005, but not violent content, *t*(1497) = −0.73, *β* = −0.03, *p* = .465, did significantly impact aggression after playing. Overall, Study 2 did not provide evidence for H2. Means for aggressive feelings before and after playing violent and nonviolent games are visualized in Figure 1.

As in Study 1, participants exhibited more aggressive feelings after playing any video game than before, *t*(2973) = 3.00, *β* = 0.08, *p* = .003. As the interaction of time and violent content was not significant, *t*(2972) = −0.40, *β* = −0.02, *p* = .686, violent content did not account for the increase in aggressive feelings. Further analyses showed that the increase in aggressive feelings was again related to competitive content, *t*(653) = 4.50, *β* = 0.03, *p* < .001, and game achievement, *t*(2168) = −9.73, *β* = −0.10, *p* < .001.

Analyses for H3 were conducted on the sample of the 124 individuals who participated in all three survey phases and who indicated at least for one session that they had played a violent video game. Furthermore, only those sessions were included in the analysis in which participants indicated that they had played a violent game. As a random effect we included sessions and as fixed predictors, we added mood after playing and aggressive feelings after playing as control variables. The outcome variable was the belief in the cathartic effect of playing violent video games. This procedure allowed to examine whether the previously found relations between mood after playing and believing in the cathartic effect would hold when controlling for aggressive feelings. As hypothesized, results showed that, when aggressive feelings after playing were included in the model, the impact of mood after playing on the belief in the cathartic effect was still significant, *t*(653) = 3.24, *β* = 0.14, *p* = .001. In contrast, reported aggressive feelings after playing did not have a significant impact on the belief in the cathartic effect, *t*(659) = 0.85, *β* = 0.08, *p* = .393.

### 3.3 Discussion

As in Study 1, we found clear empirical evidence for H1: Participants that played a violent game and were in a better mood were the ones who believe most strongly in the cathartic effects of violent video games. Again, the relationship held when controlling for sex, session, and mood before playing. Extending Study 1, we examined whether the positive relation between the player's improved mood after playing a violent game and the belief in catharsis would hold when controlling for the participant's actual level of aggressive feelings. Indeed, results showed that even when controlling for the player's level of reported aggressive feelings, the impact of a better mood after playing on the belief in the cathartic effect of playing violent video games was still significant. Interestingly, the player's actual level of aggressive feelings after playing was not significantly related to the belief in the cathartic effect of violent video games. It thus appears that the players' improved mood more than their level of aggressive feelings predicts to what extent they believe that playing violent games reduces aggressive impulses. In contrast to Study 1, neither overall aggressive feelings nor the level of aggressive feelings before or after playing was significantly related to the violence of the game.

## 4 GENERAL DISCUSSION

Violent video game play typically improves the player's mood (Bowman & Tamborini, 2015; Fleming & Rickwood, 2001; Russoniello et al., 2009). At the same time, playing video games has been shown to increase aggression (Anderson et al., 2010; Greitemeyer & Mügge, 2014; Prescott et al., 2018). Yet, in particular, among players of violent video games (Greitemeyer, 2014), there is the widespread belief that violent video game play cleanses from aggressive impulses. Given the mood‐enhancing effect of violent video game play, we hypothesized that the player's improved mood after playing would be positively related to the belief in the cathartic effect of violent video game play. Results of both studies fully support our hypothesis: the better the player's mood was after playing a violent video game, the more the player believed in the cathartic effect of playing a violent video game. This result supports our reasoning that when individuals are in a better mood after playing a violent video game, they misinterpret the improved mood as a reduction of aggressive feelings and erroneously believe that playing decreased their level of aggression. Thereby, the present research offers a possible explanation why, despite disconfirming evidence, habitual violent video game players believe that violent video game play lowers aggression.

It is noteworthy that the effect of experiencing a better mood after playing a violent video game more than the participant's level of aggressive feelings was predictive of the belief in the cathartic effects of violent video game play. In fact, whereas the effect of improved mood after playing on the belief in catharsis was still significant when controlling for aggressive feelings after playing, the effect of the participant's level of aggressive feelings was not. Overall, it appears that habitual violent video game player feel better after playing a violent video game and because of that, they erroneously believe that violent video game play makes the players less aggressive.

A secondary aim of our research was to examine the relationship between violent video game play and aggressive feelings in the players' own natural habitat. In Study 1, as expected, there was a significant relationship between violent video game play and aggressive feelings. Follow‐up analyses showed that players of violent video games tended to feel more aggressive after playing than were players of less violent games. In contrast, the player's level of aggressive feelings before playing was not significantly related to the level of violent content of the video game that was subsequently played. These results suggest that violent video game play increases the player's level of aggressive feelings, whereas gamers who feel aggressive are no more likely than others to choose a violent video game. However, Study 2 did not replicate this pattern of results. Hence, more research is needed to examine how violent video game play and the player's level of aggressive feelings interact in the player's daily life.

In both studies, participants reported to feel more aggressive after video game play than before. That is, any (not only violent) video games led to increased levels of reported aggressive feelings. Whereas violent content did not account for this effect, we found that competitive content and low game achievement were significantly related to the effect that all video games increased aggressive feelings. Previous research has repeatedly shown that competitive video games increase the player's level of aggression (e.g., Adachi & Willoughby, 2011). Likewise, the video game's outcome (i.e., winning vs. losing) has been found to be a predictor of postgame aggression (e.g., Breuer et al., 2015). In this regard, it is important to note that only habitual video game players could participate in our studies (as we surveyed participants before and after video game play in their natural habitat). Because not only video game violence but also other game dimensions, such as competitiveness and low achievement, have been shown to be associated with increases in the player's level of aggression, it is reasonable to assume that video game players, in general, tend to be more aggressive than individuals who do not play video games. Moreover, because nonplaying individuals could not participate, the range of the video game violence exposure scores was decreased. Overall, the issue that only habitual video game players participated may have contributed that the relationship between video game violence and the player's level of aggressive feelings was only small in its magnitude.

It is noteworthy that we relied on the participant's self‐reported level of aggressive feelings and avid violent video game players might be motivated to misreport their actual level of aggressive feelings after violent video game play. In fact, a study by Bender et al. (2013) showed that participants who highly identified themselves as players of video games reported less aggression on a transparent aggression measure than lowly identified players. In contrast, when a nontransparent aggression measure was used, players were indeed more aggressive after playing a violent game. Although we tried to measure aggressive feelings with less transparent adjectives, most participants should have been aware that their levels of aggressive feelings were assessed and some players might have given biased reports of their aggressive feelings. As pointed out by Gentile (2013), habitual violent video game players advocate the cathartic effect of violent video games, because they do not want to admit to themselves and others that what they are doing has harmful effects. The finding that violent content did not explain the increase in aggressive feelings of playing a video game may also be a result of the misinterpretation of mood enhancement as H1 suggests. Given that playing violent video games improves the player's mood and that the mood‐enhancing effect is related to the belief in the cathartic effect, habitual violent video game players may mistakenly rate their feelings as less aggressive directly after playing.

In line with mood management theory (Zillmann, 1988), participants were in a better mood after playing than before playing. Previous studies found that violent as well as nonviolent video game play is associated with a better mood (Villani et al., 2018). In line with these findings, we found that violent and nonviolent games lead to similar mood improvements. Overall, playing a self‐chosen video game has a beneficial impact in that it improves the player's mood.

## 5 THEORETICAL IMPLICATIONS, LIMITATIONS, AND STRENGTHS

In terms of theoretical implications, our studies provide a critical test of the opposing predictions derived from the GAM and catharsis theory. As noted in the introduction, whereas the GAM assumes that violent video game play increases aggressive feelings (among other internal states) that then instigates aggressive behavior, catharsis theory assumes that violent video game play decreases aggressive effect that then reduces aggressive behavior. We found modest support for the predictions derived from the GAM (a significant relationship between violent video game and aggressive feelings in Study 1, a finding that was not replicated in Study 2), whereas we found clear support against catharsis hypothesis. Most importantly is our finding that the belief of the cathartic effect of violent video game was more affected by the player's improved mood rather than the player's actual level of aggressive feelings.

Moreover, the present research provides the first empirical evidence that because playing a violent video game improves the player's mood, players are more likely to believe that aggression is reduced by playing. This result is in line with mood management theory (Zillmann, 1988), suggesting that playing (violent) video game leads to mood improvements. Although the catharsis hypothesis has been scientifically disproved many times (e.g., Allen et al., 1995; Geen & Quanty, 1977; Gentile, 2013), empirical evidence shows that especially violent video game players still believe in cathartic effects (Greitemeyer, 2014). The present studies' results are in line with previous findings as violent video game play was positively related to the belief in the cathartic effect. Beyond this replication, we found first empirical evidence that a positive correlation between believing in catharsis and mood‐enhancing effects of violent video games exists. Thereby, our results indicate how implications of mood management theory can play a substantial role for the prominence of the catharsis hypothesis.

The main limitation of the present research is that due to the correlational design, no causal implications can be drawn from the present research. Therefore, it is unclear whether the player's mood improvement indeed has a causal impact on the belief in the cathartic effect of violent video games. Given the longitudinal nature of the data, however, the present studies extend correlational studies by measuring aggressive feelings directly before and after playing.

Due to the naturalistic design of the present research, the assessment of aggressive feelings needed to be short as participants were asked to fill out several questionnaires a day. Therefore, aggression was assessed in the form of aggressive feelings measured by self‐report. As noted above, self‐report measures have important limitations (Bender et al., 2013). Future studies are needed to employ less transparent ways to assess aggression in naturalistic settings.

Another limitation is that all game content aspects were also assessed via self‐report. This procedure is often used in correlational studies (e.g., Anderson & Dill, 2000; Greitemeyer & Sagioglou, 2017), but it is conceivable that how a habitual video game player perceives a violent video game differs from how the same game is perceived by people that do not typically play those games. With regard to how violent the video game is perceived, for example, it may be that habitual violent video game players and people who only play violent video games every now and then have different thresholds so that the very game is perceived to be less violent by the habitual violent video game players. However, self‐report ratings of video game violence do correlate highly with ratings from objective sources (e.g., Gonzalez & Greitemeyer, 2018).

With regard to self‐report measures, we should note that we employed a repeated‐measures design in that participants reported on their level of aggressive feelings before and after video game play. This is important because people, in general, tend to be consistent and give the same responses on the “after” measures as they did on the “before” measures, thereby minimizing true effects.

According to the GAM (Anderson & Bushman, 2018), violent video games have an impact on aggressive behavior via the player's internal state consisting of cognition, affect, and physiological arousal. In the present research, we focused on the player's level of aggressive affect, as the catharsis hypothesis is about expressing, and thereby reducing, aggressive feelings. Nevertheless, future research on catharsis beliefs may include measures of aggressive cognitions and physiological arousal as well.

It should be kept in mind that results are not generalizable to the general population as only habitual video game players were examined. However, habitual violent video game players are those who most likely believe in the cathartic effect of playing violent video games (Greitemeyer, 2014). Nevertheless, our reasoning that the player's mood improvement after playing accounts for the belief in catharsis cannot account for why individuals who do not play violent video games believe that doing so would reduce aggressive inclinations. Future work is thus needed to address why some non‐players are also convinced of the cathartic effect of playing violent video games.

Concerning the sample, the majority of the participants were students and therefore the samples are not representative of the overall population. Furthermore, participants were relatively young, and more males than females participated. Future studies are needed to examine whether the results of the present studies generalize to different age groups as well as other cultures and populations.

Even though the present research has important limitations, it also comprises major advantages compared with previous studies. Whereas previous research was often conducted under laboratory conditions, the present research examined the effects of video game play in the participant's own habitat. (Although lab aggression studies tend to replicate well in real‐world settings, e.g., Anderson & Bushman, 1997). In comparison with laboratory studies, ESMs allow examining impacts of events in natural and spontaneous contexts. They enhance the external validity of results (Moreno et al., 2012) and reduce the likelihood of mistakes due to retrospection, as the amount of time between an experience and the assessment of the experience is minimized (Bolger et al., 2003). Thus, the present studies complement previous experimental studies examining the impact of violent video game play under laboratory conditions. Another strength of the present research was the relatively large sample size for experience sampling studies. Relatedly, given that the experience sampling approach requires participants to respond to the questionnaires multiple times, our findings rely on large numbers of data and hence statistical power is relatively high (compared with experimental and correlational studies).

Although abundant research on the catharsis hypothesis has been conducted, it remained vague why people believe that violent video game play makes the players less aggressive. One idea lied in cognitive dissonance reduction (Gentile, 2013) that habitual violent video game players do not want to admit that what they are doing has a harmful impact on others. Our studies complement this approach by showing that when habitual violent video game players are in a better mood after playing, they simultaneously consider themselves as less aggressive and thus believe in the cathartic effects of playing. Importantly, however, although the experienced improved mood after playing might be real, this comfortable feeling is misinterpreted as a reduction of aggression.

## 6 CONCLUSION

The present research found first empirical evidence for the reasoning that people believe in cathartic effects of acting out violence in video games, because playing has a positive impact on the player's mood. Thereby our studies are the first to show substantial connections between mood management theory and the belief in catharsis. Moreover, the present research is the first to investigate the immediate effects of violent video game play with a representative sample size in the player's own habitat and presents a foundation for future studies to examine video game effects in more naturalistic settings. Finally, we revealed the first evidence that the gap between studies showing an aggression increase after violent video game play and the habitual violent video game players' perception of a reduction in aggression is the result of a misinterpretation of being in a better mood after playing. Violent video game play does have beneficial effects for the player. However, although the players are in a better mood after playing, their anger is not vented.

## CONFLICT OF INTERESTS

The authors declare that there are no conflict of interests.

## Data Availability

The questionnaires and data for both studies are openly available in the Open Science Framework: DOI 10.17605/OSF.IO/6CRX4. The data that support the findings of this study are openly available in OSF at https://osf.io/6crx4/?view_only=b41deda62e334e2581438e7a9caa529c.

## ENDNOTES

1 When we tested the model without the covariate sex, the interaction effect of game violence and mood after playing remained significant, *t*(1441) = 2.90, *β* = 0.18, *p* = .004. When the main effect for mood after playing was included, neither the main effect, *t*(1446) = 1.62, *β* = .08, *p* = .106, nor the interaction with violent content, *t*(1446) = 0.49, *β* = 0.01, *p* = .622 were significant.

2 As in Study 1, we tested the model without the covariate sex. The interaction between game violence and mood after playing remained significant, *t*(1350) = 4.69, *β* = 0.10, *p* < .001. When the main effect for mood after playing was included, the main effect was significant, *t*(1346) = 3.02, *β* = 0.25, *p* = .003. In contrast, the interaction was not significant, *t*(1345) = −0.92, *β* = −0.05, *p* = .358.
